# Disease Ecology: Community Structure and Pathogen Dynamics

**DOI:** 10.3201/eid1301.061342

**Published:** 2007-01

**Authors:** A. Townsend Peterson

**Affiliations:** * University of Kansas, Lawrence, Kansas, USA

The disciplines of community ecology and epidemiology treat complex interactions among species, so a synthesis and integration of the 2 fields are long overdue. Because each field has insights and inferences to offer to the other, such an integration could be mutually beneficial and yield important steps toward a predictive and profound understanding. This book links an interesting framework for analyzing species’ interactions (chapter by R. Holt and A.P. Dobson) with a series of case studies regarding many host-pathogen systems, including both well-known and more novel examples. As such, this volume is a ripe field for taking the first steps toward a synthesis.

Several of the case studies are nothing short of fascinating. For example, the studies of microbial communities in ticks (chapter by K. Clay et al.) and mosquito blood meal sources as indicators of arbovirus hosts (chapter by R.S. Unnasch et al.) are impressive demonstrations of the power of melding new molecular tools with more classical epidemiologic studies. Likewise, the studies of Nipah and Hendra viruses (chapter by P. Daszak et al.) and plague (chapter by C. Ray and S.K. Collinge) offer interesting views into complex disease transmission systems. Although a parallel chapter summarizing the complex community and environmental interactions underlying hantavirus transmission would have been a nice complement, the biggest shortfall is that few of the chapters manage to link strongly to the theoretical ecologic framework offered in the chapter by R. Holt and A.P. Dobson.

More generally, the book is attractively composed and appears to be bound well and printed on quality paper. For the size and content, though, the price is quite high—I suspect that this volume will be a valued addition to any library but is perhaps unlikely to be purchased by many people. This book will, I hope, be a first step toward a new synthesis of 2 seemingly distant but intimately related fields of inquiry, and at the very least represents an intriguing compendium of well-developed case studies of the complexities of disease systems.

